# Investigating the effects of Argireline in a skin serum containing hyaluronic acids on skin surface wrinkles using the Visia^®^ Complexion Analysis camera system for objective skin analysis

**DOI:** 10.3205/iprs000179

**Published:** 2023-10-31

**Authors:** Helga Henseler

**Affiliations:** 1 Klinik am Rhein, Klinik für Plastische und Ästhetische Chirurgie, Düsseldorf, Germany

**Keywords:** Argireline, wrinkles, skin serum with hyaluronic acid, Truskin Age®, skin surface features, Visia® complexion analysis camera system, objective measurements

## Abstract

**Objective:**

To analyze the effects of Argireline on skin surface wrinkles using the Visia^®^ camera system developed by Canfield Scientific Inc., U.S.A., for facial image capture.

**Method:**

Nineteen female participants were recruited from a plastic surgery clinic. Initial facial images captured the left, front, and right sides of the participants’ faces, which were documented as timepoint one. Following this, the participants immediately began to apply a facial skin serum containing triple hyaluronic acids produced by CNC cosmetic GmbH, Philippsburg, Germany. The serum was applied once in the morning and once in the evening. Participants received two identical containers labeled L for left and R for right, with each container to be used on the corresponding facial side, particularly around the eye area. One container contained Argireline, a synthetic hexapeptide, which previously was deemed to be a biosafe alternative to botulinum neurotoxin. The study was conducted as double-blind; neither the participants nor researchers knew which of the two containers contained Argireline. Participants were allowed to use their own cosmetic products throughout the study. After four weeks, the participants returned to have their faces recaptured using the Visia^®^ camera, which was documented as timepoint two. The absolute scores of the wrinkles were noted, and results on both sides of the face were calculated and compared. The “TruSkinAge^®^” measurement provided by the Visia^®^ camera was reviewed for each face side. Results between both time points and both sides of the face were compared. After the data analysis was complete, the company was contacted to determine which container contained Argireline.

**Results:**

Nineteen participants returned for facial image capture. There were no significant adverse events, allergic reactions, or skin irritations. The investigation revealed that the wrinkle score slightly decreased for the right and left side of the face following four weeks of serum application. However, this decrease was not significant (p>0.05) based on the Wilcoxon matched pairs tests for the wrinkle scores (right side p=0.060 and left side p=0.176) and Truskin Ages^®^ results (right side p=0.096 and left side p=0.489).Comparing the data from the right side with that from the left side of the face revealed that neither demonstrated a significant reduction in wrinkle score (p=0.829) or Truskin Ages^®^ results (p=0.804). Argireline was included in the serum applied to the right side of the face. However, no statistical significance was seen in the results on this side of the face indicating any possible effects.

**Conclusion:**

Wrinkle scores and Truskin Ages^®^ results were observed to decrease non-significantly following the application of a skin serum involving hyaluronic acid. The Visia^®^ imaging method was used to analyze the data objectively. Differences between both sides of the face that were treated with and without Argireline were not statistically significant. Therefore, the effect of Argireline was not proven. While Argireline presented with low toxicity, its efficacy was found not to be significant. Therefore, it is not deemed to be an alternative treatment to botulinum toxin.

## Zusammenfassung

**Ziel:**

Objektive Analyse der Effekte von Argireline auf Falten der Hautoberfläche durch Anwendung der Visia^®^ Kamera der Firma Canfield Scientific Inc., U.S.A., für Bildaufnahmen des Gesichtes.

**Methode:**

Neunzehn weibliche Freiwillige wurden aus einer Plastisch-Chirurgischen Klinik rekrutiert. Initiale Bildaufnahmen der Gesichter von links, vorne und rechts wurden aufgenommen, was als Zeitpunkt Eins dokumentiert wurde. Danach starteten die Freiwilligen umgehend mit der Anwendung eines Hautpflegeserums, welches dreifache Hyaluronsäuren für das Gesicht enthielt, produziert durch die CNC cosmetic GmbH, Philippsburg, Deutschland. Das Serum wurde einmal morgens und einmal abends angewendet. Die Teilnehmer erhielten zwei identische Töpfchen, die mit L für links und R für rechts bezeichnet wurden; entsprechend sollten sie auf der jeweiligen Seite des gereinigten Gesichtes, besonders rund um die Augenpartie, angewendet werden. Ein Töpfchen enthielt Argireline, ein synthetisches Hexapeptid, welches zuvor als biologisch sichere Alternative zu Botulinum Neurotoxin beurteilt worden war. Die Studie wurde doppelt blind durchgeführt, wobei weder die Freiwilligen noch die Forscher wussten, welches der beiden Töpfchen das Argireline enthielt. Die Freiwilligen durften zusätzlich ihre eigenen kosmetischen Produkte während der Studie benutzen. Nach vier Wochen kamen die Teilnehmer zurück, um das Gesicht erneut mittels der Visia^®^ Kamera fotografiert zu bekommen, was als Zeitpunkt Zwei vermerkt wurde. Die absoluten Scores der Falten wurden notiert und die Ergebnisse der beiden Seiten des Gesichtes berechnet und verglichen. Ebenfalls wurde das gemessene „TruskinAge^®^“, das von der Visia^®^ Kamera herausgegeben wurde, für jede Gesichtshälfte untersucht. Die Ergebnisse für beide Zeitpunkte und beide Seiten des Gesichtes wurden verglichen. Nachdem die Datenanalyse vollständig war, wurde die Firma kontaktiert, um zu bestimmen, welches Töpfchen das Argireline enthielt.

**Ergebnisse:**

Neunzehn Freiwillige kamen für die Gesichtsaufnahmen zurück. Es gab keine signifikanten Nebenwirkungen, keine allergischen Reaktionen oder Hautirritationen. Die Untersuchung ergab, dass der Faltenscore für die rechte und linke Seite des Gesichtes nach vier Wochen der Anwendung der Produkte leicht abnahm. Allerdings war die Abnahme nicht signifikant (p>0.05) gemäß des Wilcoxon-Testes für zwei abhängige Stichproben für die Faltenscores (rechte Seite p=0,060 und linke Seite p=0,176) und für die TruskinAge^®^ Ergebnisse (rechte Seite p=0,096 und linke Seite p=0,489). Beim Vergleich der Daten der rechten mit denen der linken Seite des Gesichtes ergab sich, dass keine der Gesichtshälften signifikant besser abschnitt als die andere in Hinblick auf die Reduzierung der Faltenscores (p=0,829) und der TruskinAge^®^ Ergebnisse (p=0,804). Argireline war im Serum enthalten, das auf der rechten Seite des Gesichtes angewendet wurde. Allerdings wurde keine statistische Signifikanz der Ergebnisse auf dieser Seite des Gesichtes im Hinblick auf mögliche Effekte erreicht.

**Schlussfolgerung:**

Die Scores der Falten sowie die Ergebnisse der Truskin Ages^®^ zeigten eine nicht signifikante Tendenz, nach Anwendung des Hautpflegeserums basierend auf Hyaluronsäuren zurückzugehen. Die Visia^®^ Bildgebungsmethode wurde für die objektive Datenanalyse eingesetzt. Die Unterschiede zwischen den beiden Gesichtshälften, die mit und ohne Argireline behandelt wurden, waren nicht statistisch signifikant. Daher wurde die Wirkung von Argireline nicht nachgewiesen. Während sich Argireline mit niedriger Toxizität darstellte, wurden seine Effekte als nicht signifikant ermittelt. Daher wird es nicht als alternative Behandlung zu Botulinumtoxin eingestuft.

## Introduction

The application of botulinum neurotoxins revolutionized cosmetic science due to their anti-wrinkle activity. However, their high neurotoxicity presented a problem and created a need to find alternative options that produced similar results with lower toxicity. The first scientific publication on the usage of Argireline for aesthetic improvements of wrinkles occurred twenty years ago [1]. Argireline, an acetyl hexapeptide-8 formulation cited as having properties similar to botulinum, was provided to 10 healthy females in an oil/water emulsion that contained 10% Argireline. Silicone imprints were analyzed by confocal laser scanning microscopy, and further assessments with a 10x magnifying glass were conducted. Argireline reduced wrinkle depth by up to 30% after four weeks of treatment without exhibiting toxicity, while the oil/water emulsion on its own reduced wrinkle depth by up to 10%. Argireline was deemed to be a non-toxic anti-wrinkle peptide that was a biosafe alternative to botulinum neurotoxins, albeit with a lower efficacy. 

Several further studies have investigated Argireline in aesthetic medicine [2], [3], [4]. While an early study focused on its preparation and stability within cosmetic formulations [2], later its topical usage in the treatment of blepharospasm in patients receiving botulinum toxin therapy was studied and no significant adverse effects were found [3]. Based on a baseline Jankovic tape investigation, the blepharospasm returned after 3.7 months in the Argireline group (n=12) compared to 3 months in the placebo group (n=11). However, this difference was non-significant. Nevertheless, the Argireline group demonstrated better scores, with one-third showing considerable improvement in symptom control. The topical application of Argireline was deemed a safe and promising alternative to botulinum neurotoxin.

Wang conducted a prospective, double-blind, randomized, placebo-controlled study investigating the anti-wrinkle efficacy of Argireline in 60 Chinese participants [4]. The periorbital lines were studied, with participants randomly receiving Argireline or placebo treatment twice daily for 4 weeks. The ratio of the randomization between Argireline and the placebo was 3:1. The total anti-wrinkle efficiency in the Argireline group was 48.9% based on subjective assessment. Wang used Daniell’s classification [5], a four-point wrinkle severity scale (none, mild, moderate, and severe), and Seeman’s standard [6], a five-point rating scale (from 0 to 4, based on the level of improvement). Wrinkle depth was described as being significantly reduced in the Argireline group (p<0.01). The objective assessment was conducted using silicone skin replicas of the treated areas, analyzed via a wrinkle analysis apparatus (Skin-Visioline VL 650^®^; Courage + Khazaka Electronic GmbH, Germany), and processed using confocal laser scanning microscopy. All roughness parameters were decreased in the Argireline group. Overall, a significant anti-wrinkle effect was postulated.

Further investigations utilizing various measurement methods to assess Argireline have been published. However, none have used the high-resolution Visia^®^ camera system, an objective measurement tool for skin analysis. This camera system, developed by Canfield Scientific Inc., New Jersey, USA (https://www.canfieldsci.com/), visualizes several skin aspects using various flashes and enables the objective analysis of wrinkle number and intensity using three different measurement methods [7]. There is a paucity of independent publications investigating the usage of the Visia^®^ camera system, although the system was recently reviewed to enable objective outcome studies [8]. However, to date, no independent clinical research study has investigated Argireline and its botulinum toxin similar properties using this system.

## Objective

The objective of this study was to objectively analyze the effects of topically applied Argireline on skin surface wrinkles, using a hyaluronic acid-based serum to deliver the Argireline and the Visia^®^ camera system for facial image capture. 

## Method

Nineteen female participants were recruited from a plastic surgery clinic via verbal and digital invitation. Information was provided via individual consultation with a consultant plastic surgeon in the clinic and using an information sheet. Verbal and written consent was obtained at the beginning of the study. In the event of any problems, participants were advised to contact the clinic. The principal investigator was available throughout the entire study. Participation was voluntary. 

The inclusion criteria for the study were females who consented to participate and agreed to apply the products according to the protocol. In contrast, females who received Botox injections in the previous 8 months or during the course of the study, had known allergies or sensitivity to the products used, presented with acute health problems or acute or chronic skin problems, or did not wish to return for the follow-up captures were excluded. 

**Figure 1:**
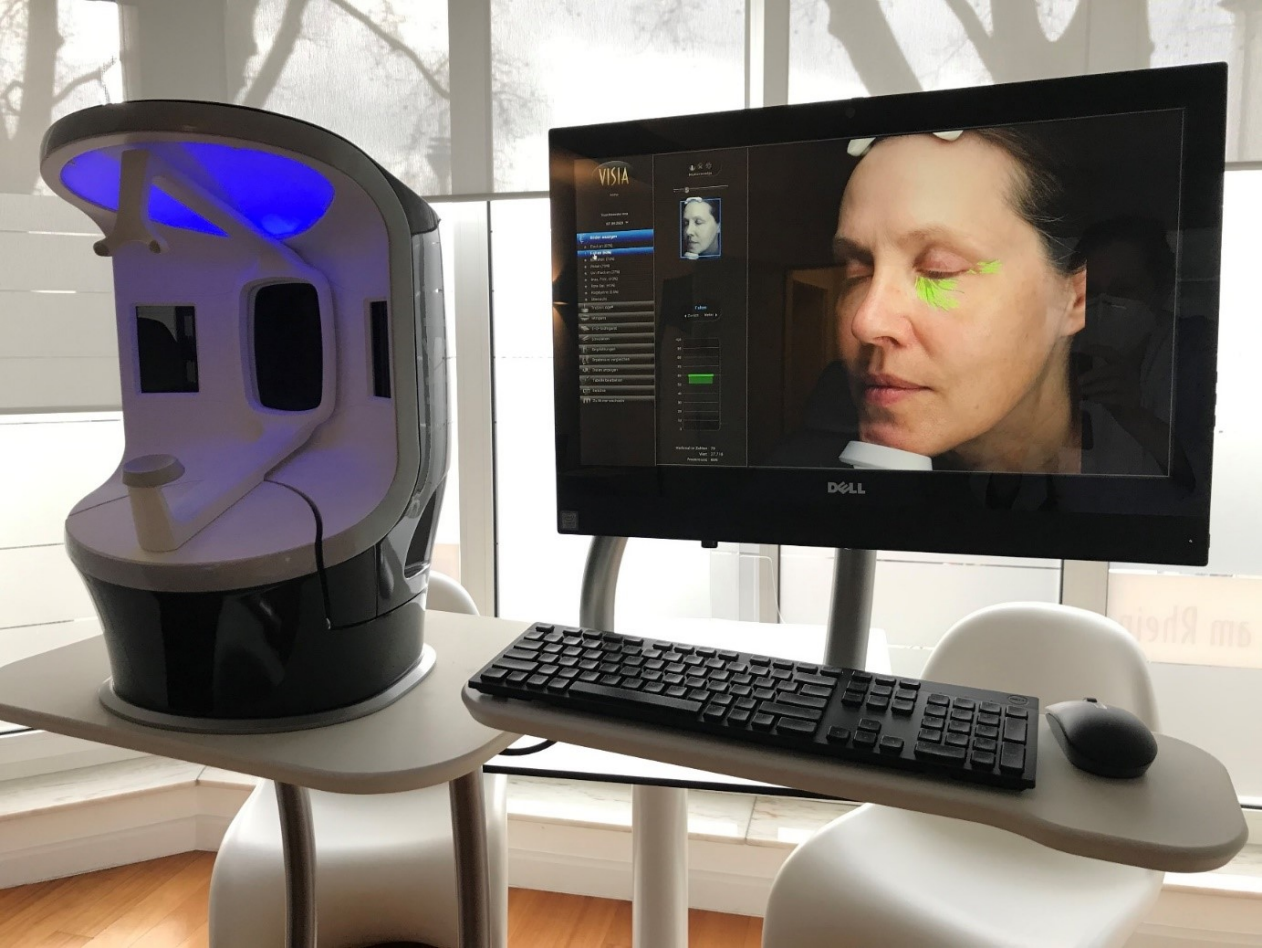
Visualization of wrinkles on the left side of the face using the Visia^®^ camera system

Initial facial captures of the left, front, and right sides of the face were conducted on clean, make-up-free skin. These images were documented as timepoint one (capture 1; C1). Facial capture was conducted using the Visia^®^ camera system (Figure 1 (Fig. 1)).

**Figure 2:**
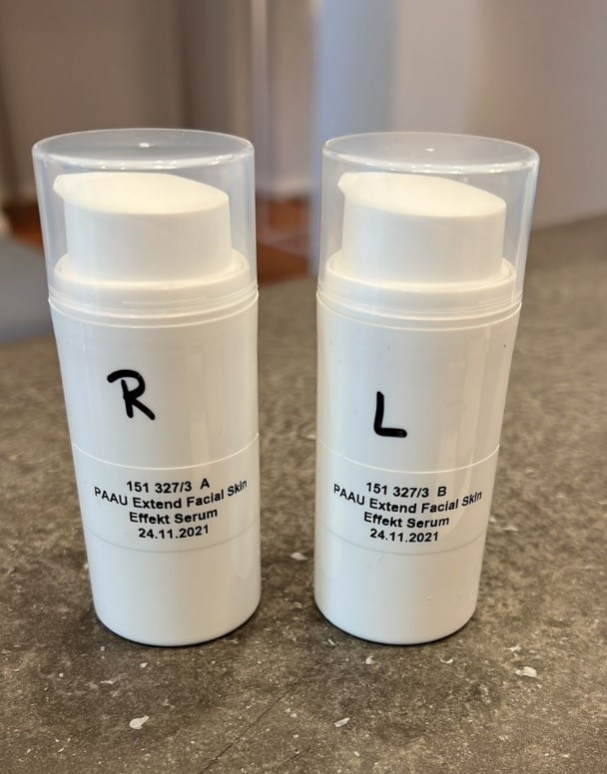
The skin serum was provided in two identical containers to be used on the right and left sides of the face.

Following this, participants immediately began to apply a facial skin serum containing triple hyaluronic acids produced by CNC cosmetic GmbH, Philippsburg, Germany, for use by PAAU, a German medical company. Participants were instructed to apply the serum once in the morning and once in the evening on clean, make-up-free skin, particularly focusing on the eye area. The serum was provided in two identical containers, labeled L for left and R for right, to be used on the corresponding side of the face (Figure 2 (Fig. 2)).

One container contained Argireline, the other did not. Argireline application was only topical; no injections were involved. The study was conducted as double-blind; neither the participants nor the researchers knew in which container the Argireline was. Participants were allowed to use their own cosmetic products in addition to the serum throughout the study. 

After four weeks of application, participants’ faces were recaptured with the Visia^®^ camera. These images were documented as timepoint two (capture 2; C2). The effect of Argireline on skin surface wrinkles was investigated objectively using the Visia^®^ camera analysis, and the results from both sides of the face were compared. The objective data captured by the Visia^®^ camera were recorded as “absolute scores,” for which the software counted the number of pixels within the area analysis. A decrease in the number of pixels (i.e., absolute score) was considered an improvement. The absolute scores indicated the magnitude and intensity of wrinkles present on the skin. The TruSkin Age^®^ was measured using the camera system for both sides of the face. These results were compared with the true biological age of the corresponding participant. 

The Wilcoxon matched pairs test was used for intra-individual comparisons, such as those between the two time points (C1 and C2), as well as for comparisons between both sides of the face (right and left) [9]. Statistical analyses were conducted using the BiAS program for Windows [10]. A p-value smaller than 5% (p<0.05) was considered statistically significant.

Clinical data collection occurred between May and October 2022. Following data analysis, the company that provided the serums was contacted to determine which container had Argireline in it. They confirmed it was the right container.

## Results

### 1 Descriptive data overview for all variables

The descriptive parameters of all continuous variables for all participants are shown in Table 1 (Tab. 1). All nineteen participants returned for follow-up facial image capture. Data from C1 and C2 images are presented. The age range of the participants was 24–68 years, with a mean age of 51.1 years and a standard deviation of 10.4 years. 

The mean absolute C1 score was 30,043±9,771 pixels for the right side of the face versus 31,860±8659 pixels for the left side. At C2, the mean absolute score was 27,653±7,753 for the right side and 29,584±9,097 pixels for the left side. Therefore, the mean wrinkle scores decreased from C1 to C2, with the right side decreasing by 2,390 pixels and the left side by 2,276 pixels.

The discrete variables demonstrated different frequency distributions, with “positive” indicating fewer wrinkles and lower estimated age when comparing the data from C1 and C2 (Table 2 (Tab. 2)).

Regarding wrinkle scores, positive developments (i.e., a lower wrinkle score at C2 compared to C1) were seen in 63.2% (12 out of 19) of participants for the right side and in 73.7% for the left side (14 out of 19). Regarding the combined scores for both sides of the face, 68.45% of participants demonstrated an improved wrinkle score. Therefore, more than two-thirds of participants demonstrated a wrinkle score reduction.

When reviewing the data in Table 2 (Tab. 2), it was noted that the data provided for the absolute wrinkle scores were more detailed than for the calculated Truskin Ages^®^. 

Regarding the Truskin Age^®^ data, several participants showed no change in their Truskin Ages^®^ between C1 and C2, meaning these participants did not fall in either the “yes” or “no” category regarding positive development. Therefore, a third category had to be developed (neutral) to compare Truskin Age^®^ data (Table 3 (Tab. 3)). 

The number of participants who exhibited a positive development in their Truskin Age^®^ (i.e., C2 was smaller than C1) was higher than the number who demonstrated a negative development for both sides of the face (right side: 42.1%, versus 21.1%, respectively; left side: 47.4% versus 31.6%, respectively).

**Figure 3:**
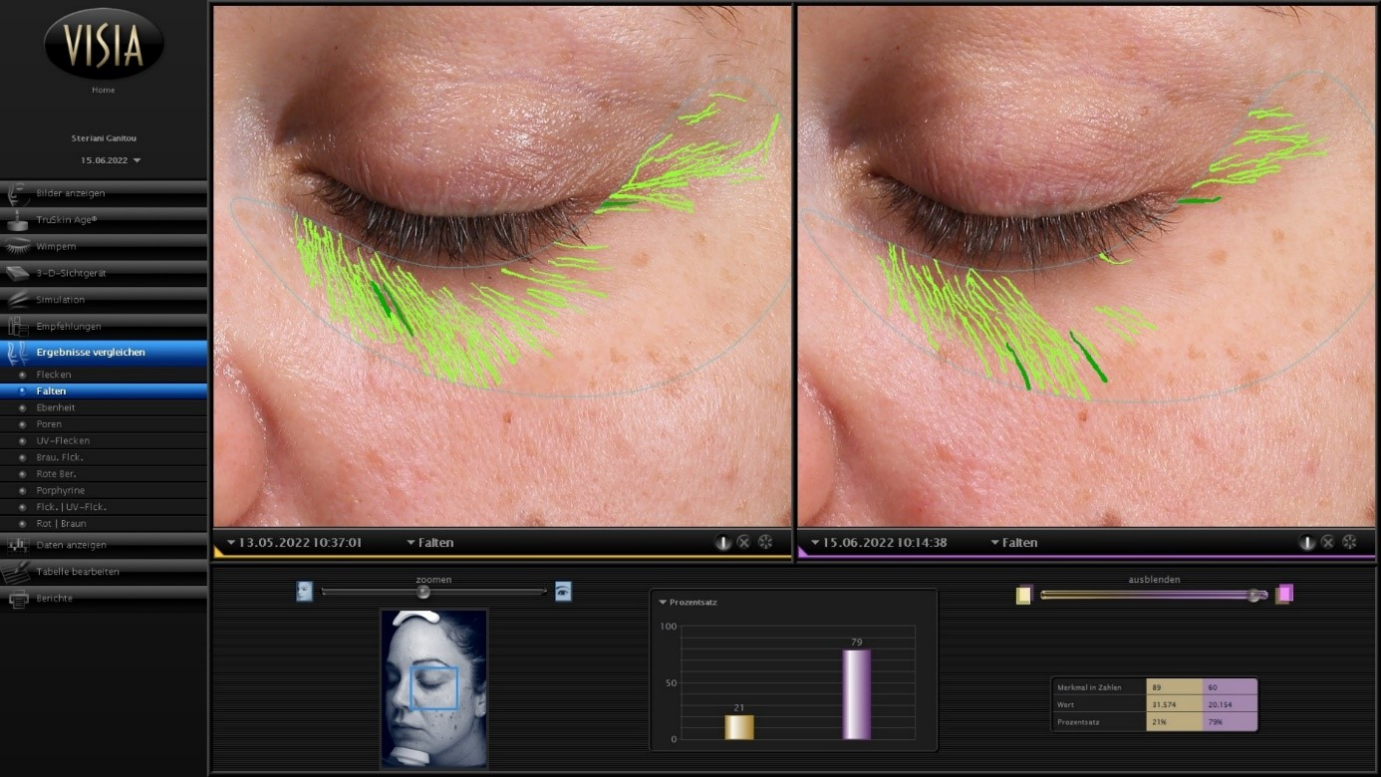
Example of a participant demonstrating a positive wrinkle score development from C1 (left) to C2 (right)

A positive or neutral development in Truskin Age^®^ was seen in 79% of the participants on the right side of the face and in 68% on the left side. Therefore, the absolute wrinkle scores and Truskin Age^®^ scores tended to decrease between C1 and C2. For illustration purposes, an example of a positive development in the wrinkle score (i.e., a score reduction) of a study participant is shown in Figure 3 (Fig. 3). 

### 2 Statistical comparison of C1 and C2 regarding wrinkle score and Truskin Age^®^ for both sides of the face

A statistical comparison between C1 and C2 was conducted in terms of the absolute wrinkle scores and Truskin Ages^®^ was conducted for both facial sides (Table 4 (Tab. 4)). 

There was a higher reduction in the mean absolute wrinkle score on the right side (30,043 versus 27,653) compared to the left (31,860 versus 29,584); however, neither of these reductions was statistically significant. Regarding the Truskin Ages^®^, the C2 means had reduced by approximately half a year for both sides of the face compared to the C1 means (right side: 52.47 versus 51.95; left side: 52.47 versus 52.05). 

When looking at the medians of the data, a different picture emerges compared to the means. The median absolute wrinkle score for the right side reduced from 29,387 at C1 to 29,345 at C2, a total reduction of 42 points. For the left side, the median reduced from 32,624 at C1 to 28,678 at C2, a total reduction of 3,946 points. 

**Figure 4:**
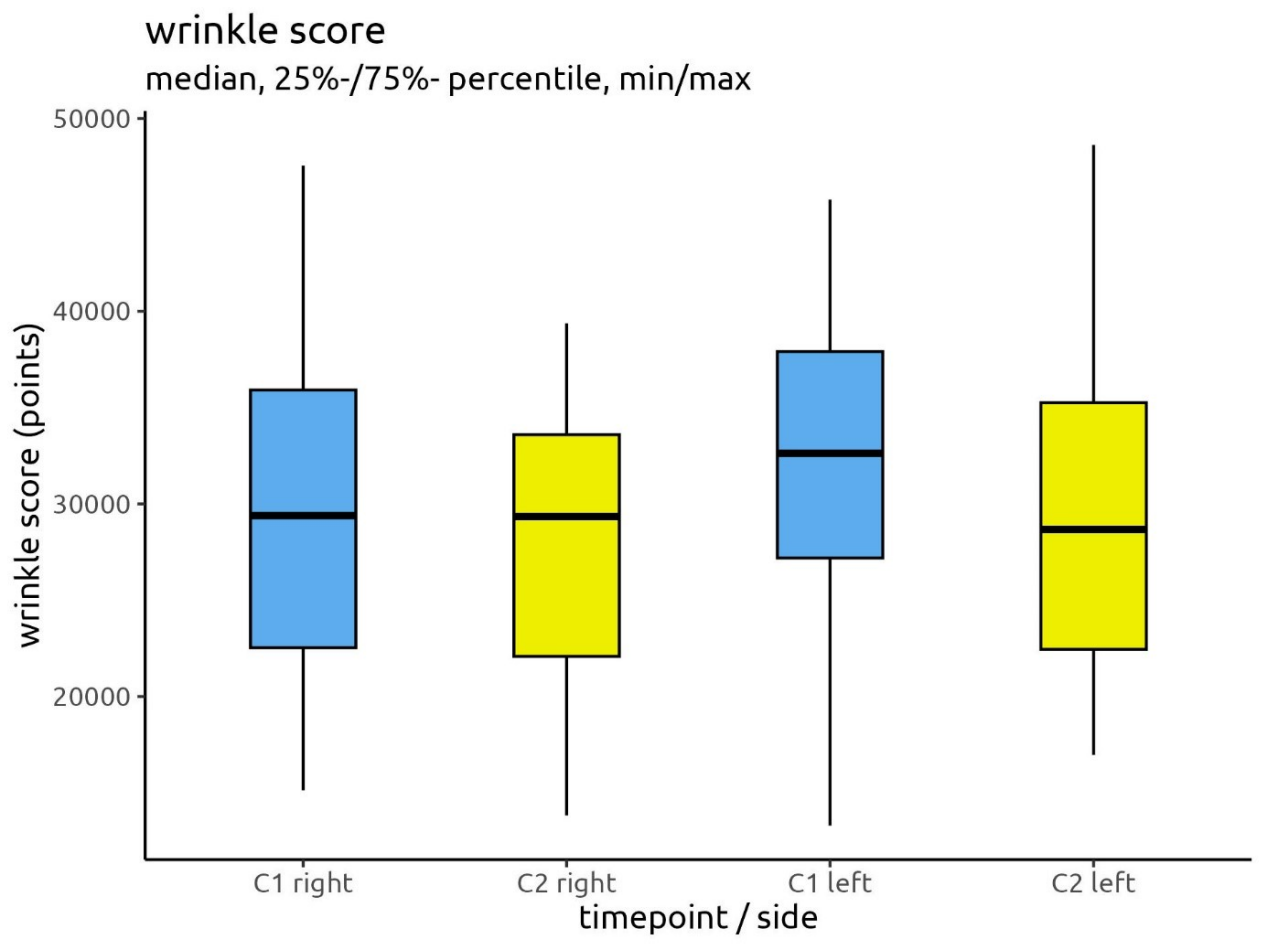
Reduction of wrinkle scores between C1 and C2 for the right and left sides of the face

Figure 4 (Fig. 4) demonstrates the reduction in wrinkle scores. The medians are displayed centrally as horizontal black lines.

**Figure 5:**
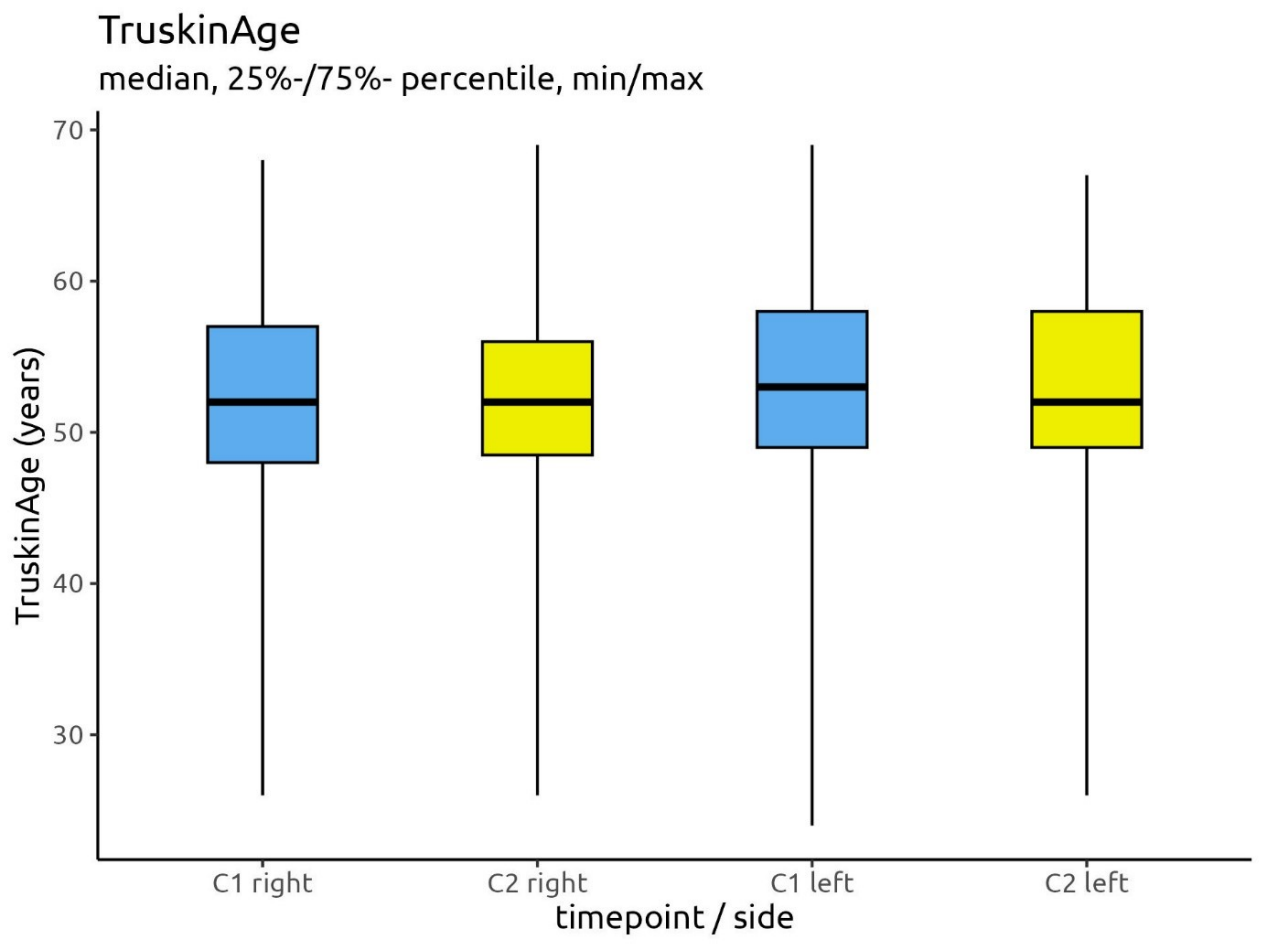
Differences in Truskin Ages^®^ between C1 and C2 for the right and left sides of the face

When looking at the Truskin Age^®^ scores, the medians demonstrated little change between C1 and C2. The median for the right side of the face was 52 years at both time points, while it was 53 years at C1 and 52 years at C2 for the left side. Figure 5 (Fig. 5) depicts the Truskin Age^®^ scores.

A Wilcoxon matched pairs test was carried out to determine whether the differences in wrinkle scores and Truskin Ages^®^ between C1 and C2 for each side of the face were significant. Neither the wrinkle scores (right side: p=0.060; left side: p=0.096) nor the Truskin Age^®^ scores (right side: p=0.176; left side: p=0.489) demonstrated statistically significant differences (Table 4 (Tab. 4)).

### 3 Statistical comparison of the right and left sides of the face regarding their calculated differences

Based on the direct comparisons of C1 and C2, the individual differences between C1 and C2 were derived from the wrinkle score and Truskin Age^®^ data (Table 5 (Tab. 5)).

The mean wrinkle score differences were 2,389 and 2,276 for the right and left sides of the face, respectively. The median wrinkle score difference was 866 points for the right side and 1,012 points for the left side. These measurements revealed for each participant how large the reduction of the scores between both time points was. The same applied for the Truskin Age^®^ scores. For the Truskin Age^®^ scores, the median difference for both facial sides was 0 years. 

**Figure 6:**
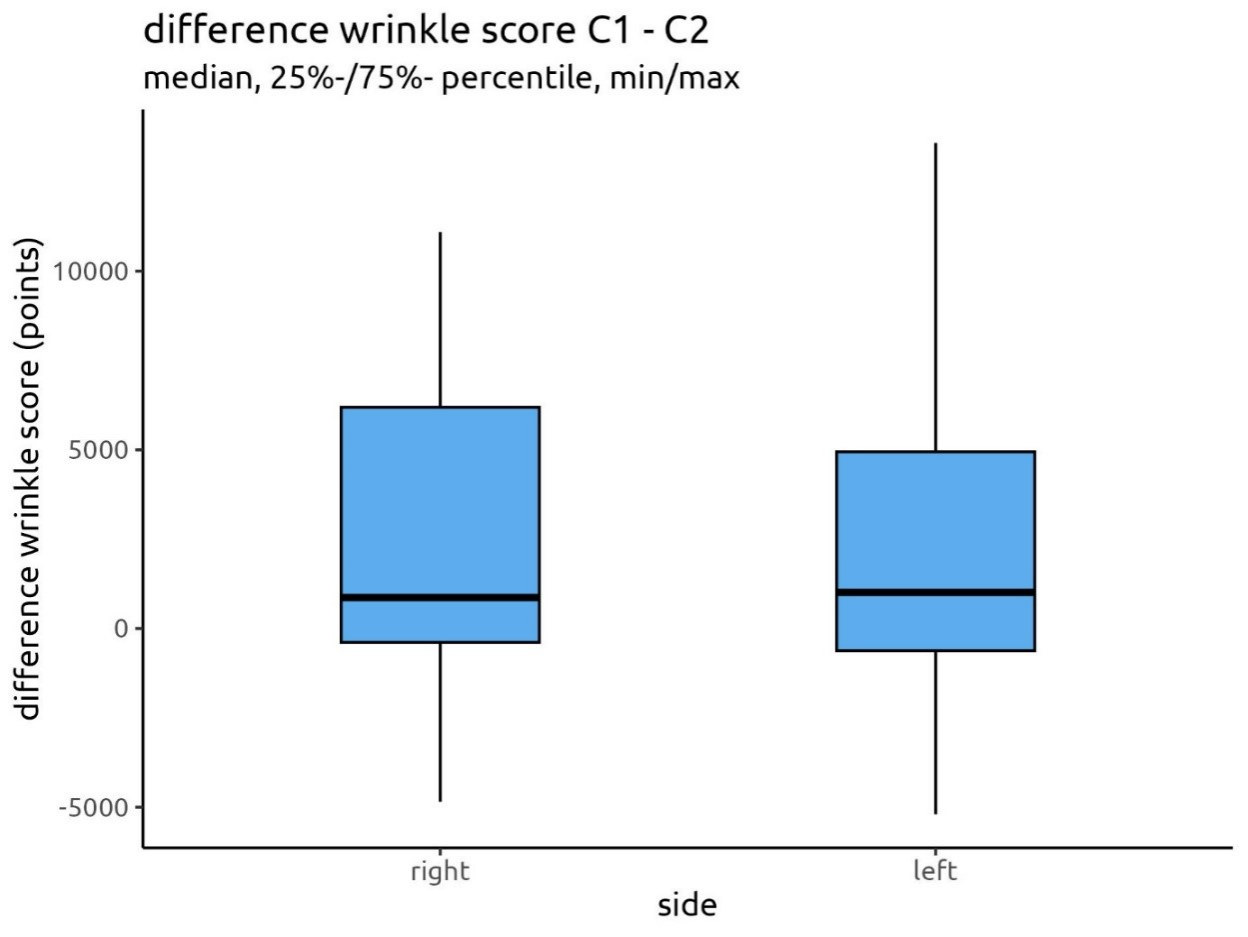
Difference in wrinkle scores between C1 and C2 for both facial sides

**Figure 7:**
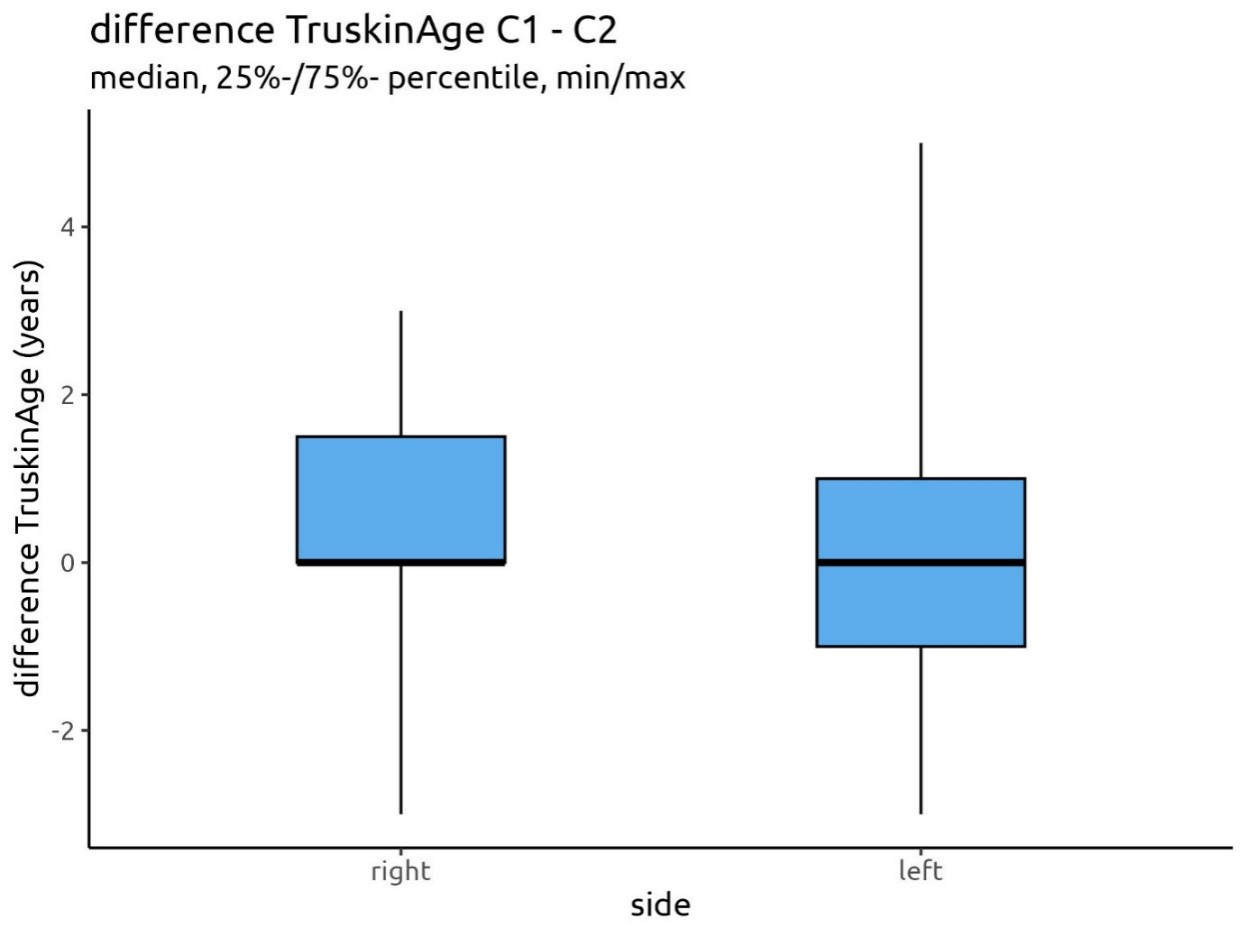
Difference in Truskin Age^®^ scores between C1 and C2 for both facial sides

Using a Wilcoxon matched pairs test, the wrinkle scores and Truskin Age^®^ scores of each facial side were compared (Table 5 (Tab. 5)). No statistically significant difference was found for the wrinkle scores (p=0.829) or Truskin Age^®^ scores (p=0.804). Therefore, neither facial side demonstrated significant improvements in either parameter. These findings are displayed in Figure 6 (Fig. 6) and Figure 7 (Fig. 7). 

Overall, there was a weak reduction in the measurements between C1 and C2, meaning there were slight improvements in the intensity and magnitude of wrinkles and Truskin Ages^®^ during the study. These positive developments were slightly stronger on the left side of the face when analyzing the median data. However, none of the differences analyzed were statistically significant (Table 5 (Tab. 5)). Therefore, Argireline demonstrated no significant effect on wrinkles or Truskin Ages^®^. 

## Discussion

This study did not prove the efficacy of Argireline, as no statistically significant results were obtained. However, objectively wrinkles and Truskin Ages^®^ appeared to decrease slightly overall in the study group. There were somewhat confusing results regarding the calculated means versus the medians, with the reduction in wrinkle scores appearing to be stronger on the right facial side when looking at the mean scores but stronger on the left facial side when looking at the medians. However, the calculations for these statistical concepts are completely different. From a statistical perspective, comparing the medians of data is preferable. While the mean is the average of all figures, calculated by dividing the sum by the number of figures included, the median is the middle figure determined when all figures are ordered numerically. Therefore, due to the risk of outliers which could significantly impact the mean value, medians are considered more representative, as they are more robust against strong deviations. The Wilcoxon matched pairs test, used for the statistical evaluation of the data, is a non-parametrical method with which pairs of data (e.g., C1 and C2) are compared to determine to what degree they differ from a purely random distribution [9]. In the end, no results regarding the efficacy of Argireline demonstrated statistical significance. 

In comparison to the first study on Argireline by Blane in 2002, the current study involved a larger number of study participants [1]. Blane’s study involved 10 participants, a sample size that is rather too small to draw any generalizable findings from. Additionally, Blane used the F-Fisher’s test with the significance level set at p<0.075, an unusually high threshold. This threshold is not normally used, as it limits the ability to rule out random errors in the evaluation of statistical significance. Like the findings of the current study, Blane concluded that Argireline had a low level of toxicity. Blane also noted the low efficacy of Argireline in comparison to botulinum toxin. Nonetheless, Blane concluded that Argireline was a biosafe alternative to botulinum toxin. In comparison, the current study found that the efficacy of Argireline was significantly lower indeed and, therefore, does not share the conclusion that Argireline is an alternative to botulinum toxin. Measurement methods have improved over the last twenty years, and while measurements using silicone imprints and magnification glasses, such as the ones conducted by Blane, were acceptable two decades ago, this study used a more modern measurement method. In fact, the current study used what is deemed the most precise system in skin surface assessment currently available, according to manufacturers.

Interestingly, a previous study investigating the use of Argireline in patients with blepharospasm also found no statistically significant effects [3]. However, a trend this study did find was that Argireline treatment lengthened the period of time before the blepharospasm reoccurred in one-third of participants. Comparatively, the current study also observed a trend where, in some participants, a measurable effect was detectable. The reasons for these non-significant results must be evaluated, and possible explanations should be discussed. Potential discussion points could include participant compliance, sample size, and unknown errors in the measurement method. A lack of skin permeability to Argireline or its overall limited efficacy should also be considered.

Another issue regarding previous research into the possible effects of Argireline is that such studies based their claims not only on a limited number of participants [1], [3] but also on subjective assessment methods [4]. A study by Wang used subjective assessments in the form of four- and five-point scales [5], [6]. Subjective assessments, however, are typically regarded as imprecise evaluation methods [11]. Larger numbers of errors are seen in subjective assessments compared to objective measurement methods [12], [13]. Therefore, Wang’s conclusion that Argireline reduced wrinkles by 48.9% must be viewed with caution. While this study claimed to be prospective, double-blind, randomized, placebo-controlled, by application of an objective assessment method only a reduction in skin roughness was described.

The current study focused on the analysis of wrinkles using the Visia^®^ camera produced by Canfield Scientific, currently viewed as the gold-standard system for visualizing the face and providing objective skin analyses. Previous studies independently examined the system and the measurement methods and skin aspects it investigates [7], [8], deeming it a useful tool for wrinkle assessment. The Truskin Age^®^ measurements that the camera enables were also of interest. 

Wrinkles are creases, furrows, or folds in the skin that increase with age, through muscular activity, as a result of sun exposure, or due to bad habits, such as smoking. The Visia^®^ camera detects wrinkles by their characteristic long and narrow shape. Interestingly, previous research has noted that the Visia^®^ camera can visualize wrinkles that cannot be seen by the naked human eye [8]. 

Another area of interest in research is the optimal method of Argireline application. In the current study, Argireline was applied topically on the skin via a serum containing hyaluronic acid. Due to the results of previous research studies, administering Argireline via injection was not considered. Previous studies have investigated the cytotoxicity of Argireline and have postulated that Argireline does not require injection [14]. The antiproliferation effect of Argireline on several human cell lines has been studied, comparing it with doxorubicin, a standard comparison compound used for assessing the cytotoxicity of unknown substances. Argireline was found to be a safe compound in comparison to doxorubicin. 

Another study stated that the transdermal delivery route offered advantages for the delivery of peptides and proteins, such as Argireline [15]. The transdermal permeation of peptides is a complex process. Iontophoresis resulted in an approximately 30-fold increase in peptide permeation relative to passive peptide permeation. This increase was influenced by a number of parameters that were thought to improve effective transdermal delivery. 

Argireline is nowadays included in a variety of cosmetic products. Therefore, its ability to penetrate the skin is of interest. The effect of different delivery vehicles has been previously examined [16]. In porcine skin, a water-in-oil-in-water emulsion was found to be a superior delivery vehicle for the increased permeation of Argireline compared to other emulsion types. This study used electron microscopy and nuclear magnetic resonance (NMR) imaging to determine this.

A discussion emerged whether peptides in cosmetics can penetrate deeper into the skin layers and stimulate biological activity. A study by Kraeling analyzed guinea pig and human cadaver skin and used hydrophilic interaction liquid chromatography with tandem mass spectrometry to measure the depth to which Argireline penetrated the skin [17]. Most Argireline remained in the stratum corneum of the epidermis, and none was detected in the dermis. Therefore, it was considered safe for use in cosmetic products. These findings are in line with those of the current study, which found no adverse effects related to Argireline use. Furthermore, the lack of statistical significance in the results of the current study is supported by Kraeling’s findings regarding Argireline remaining in the stratum corneum of the epidermis. If this is true, it explains why Argireline failed to display a similar effect as the botulinum toxin, which inhibits neurotransmitter release at the neuromuscular junction and, therefore, requires deeper penetration of the skin. However, it is important to note that the use of guinea pig and human cadaver skin by Kraeling is a limitation, as live human skin may react differently. 

Also, the use of Argireline alone (applied as a topical 10% solution), or in combination with 5% tripeptide-10 citrulline (Decorinyl^®^), or the use of Decorinyl alone, or a placebo [18] was investigated. The aim was to examine a possible synergism between the two peptides. Measurements were conducted using Skin Visioscan VC98 (Courage and Khazaka, Cologne, Germany), while transepidermal water loss (TEWL) was measured using the Tewameter TM 300 (Courage and Khazaka). TEWL significantly decreased following Argireline treatment, confirming its anti-wrinkle activity. This activity was also seen in treatment with only tripeptide-10 citrulline. When combined, the two peptides appeared to act synergistically, albeit with an unclear underlying cause. However, this study involved a small sample size (n=4) in each of the four different groups.

In contrast, the current study involved the intra-individual comparison of 19 participants treated as a homogenous group, thereby reducing the possibility of errors associated with the use of inter-individual comparisons in small sample sizes. Furthermore, the current study involved the longitudinal comparison between two time points within the same group of study participants. Therefore, there were no differences in demographic factors that could have impacted the results. While whether a comparison with a second group that used a different treatment would have been beneficial could be discussed, from a statistical perspective, drawing comparisons within the same group is preferable and more reliable, as comparisons with a separate group could be impacted due to different influencing factors between the groups. 

As the true clinical effect of Argireline remains of interest to researchers, possible modifications to its molecular structure that may enhance its effect have been studied. Specifically, modifications that aim to enhance its skin permeability have been published, as Argireline has limited permeability due to its large molecular weight and hydrophilicity [19]. One study found that a zwitterionic form and charged state hindered the skin permeation of Argireline, although it was possible to improve this permeation. Furthermore, the study assessed several modifications to determine which resulted in the biggest reduction in wrinkle formation. The authors of this study cited the findings of Wang, however without discussing the possible limitations of the study [4]. 

The topical application of Argireline within a cosmeceutical cream (Vitaeyes instant ageback) was examined in a study involving 26 participants with various conditions such as skin cancer, surgical scars, hidradenitis, and wrinkles. Investigation was conducted via photographs and investors’ clinical assessment with the help of several devices with probes pressed against the skin. The assessed skin quality parameters, including hydration, elasticity, and sebum, were found to have improved following Argireline treatment [20]. Therefore, the topical application of 10% concentrated Argireline within a cosmeceutical cream was deemed a safe and effective alternative to invasive procedures to improve the quality of life among these patients. A slight decrease in skin hydration level, a clear reduction in skin sebum level, and an increase in skin elasticity level were observed. The appearance of scars improved, as did patient well-being, which was assessed using the SF-36 questionnaire. However, this study is limited due to the limited time period it was conducted over. After the application of the cream, participants waited 5 minutes and received a gentle massage, following which the effects were observed. The improvements were stated to last for 4–8 hours. Some images before and after the treatment as well as images of a single participant’s scar development after 24 hours, 10 days, and 3 months were included in the publication; however, no further detail regarding the treatment protocol during this period was provided. 

The current study chose to investigate a homogenous group to assess the efficacy of Argireline, as opposed to the previous study by Palmieri, which involved a heterogenous study group. Notably, the study did not conclude that Argireline was effective at reducing the appearance of wrinkles; however, its topical application safety was described. Furthermore, the reported improvement in participants’ quality of life following Argireline treatment was measured using a subjective questionnaire. In contrast to Palmieri’s study, the current study involved a homogenous group of participants without prior skin conditions and used an objective measurement tool. Moreover, a standardized protocol for the application of Argireline was followed by the entire study group over four weeks. 

Other applications of Argireline have been investigated, such as its potential use in combination with a non-ablative fractional laser to improve the treatment of striae distensae [21]. In this study, 10 patients reported a subjective improvement, while the researchers reported that Argireline objectively improved the treatment of striae distensae and reduced inflammation and hyperpigmentation. However, the study population was rather small and, therefore, further investigations are needed.

The current study was conducted as double-blind and involved the topical application of Argireline twice a day in females that had not received Botox for at least 8 months prior to the beginning of the study. Cosmetics containing Argireline have attracted public interest due to their potential anti-wrinkle abilities. In fact, in some instances, this effect has been presented as confirmed based on the studies by Blane [1] and Wang [4]. It has been stated that, via the inhibition of neurotransmitter release, Argireline produces a Botox-like effect. One study investigated the possible presence of Argireline in cosmetic creams and sera by using reverse-phase liquid chromatography and tandem mass spectrometry [22]. Argireline and its oxidized form were found in several different cosmetics. It was postulated that the oxidation of Argireline may impact the analysis of its presence in cosmetic products, as well as the stability of these products. 

As with any substance, questions have been raised regarding potential complications associated with Argireline application. While several studies have reported on the safety of topical Argireline application, a study by Chen in China investigating potential post-Argireline injection complications reported differently [23]. This study described a case of *Mycobacterium* *abscessus* infection following a facial injection of Argireline in an otherwise healthy 45-year-old patient. Erythema nodules and abscesses appeared. The patient required double antibiotic therapy (clarithromycin and moxifloxacin) for 5 months. Although the facial lesions gradually subsided, some scars and pigmentation persisted. It was concluded that such infection could easily be misdiagnosed as being due to a common bacterial origin. However, there is an increasing prevalence of *M. absessus*, which has been associated with the increasing prevalence of invasive cosmetic procedures. In contrast to these findings, in the current study, Argireline was only applied topically, and no infections or adverse effects occurred. The study by Chen provided a valuable contribution to the discussion regarding the risks associated with the subcutaneous injection of cosmetic substances, particularly those carried out by untrained personnel.

Further research has investigated the potential efficacy of Argireline in a 3D-bioengineered human skeletal muscle model [24]. The muscle-relaxing properties of Argireline were evaluated in healthy and aged tissue models to assess the morphological and functional changes that occur during the aging process of muscular tissue. Results showed that the 3D-bioengineered human muscle platform was judged as useful in the assessment of these changes and presented with potential applications in biomedicine and bio-hybrid robotics. 

An investigation into the possible complications associated with periocular aesthetic treatments reported that topically administered Argireline could be a safer, albeit less effective, treatment option for periorbital rhytids compared to botulinum toxin [25]. The study reported that Argireline acts as a competitive SNAP-25 inhibitor, inhibiting the release of acetylcholine at the neuromuscular junction with lower potency and less toxicity than botulinum toxin. Furthermore, the author noted that as Argireline is considered a cosmetic, not a drug, it is not regulated by the FDA. 

Although the current study observed no complications from the topical application of Argireline, it appeared profoundly limited in its efficacy. These results are in line with the aforementioned study. Based on the current results, Argireline, however, cannot be considered a botulinum toxin alternative, as its efficacy is too low. 

## Conclusion

Using the Visia^®^ imaging method, wrinkle and Truskin Age^®^ scores were found to be reduced following the application of Argireline within a skin serum containing hyaluronic acid; however, these reductions were not statistically significant. Furthermore, there were no significant differences between the facial side treated with Argireline and the side that was not. Therefore, no effect of Argireline was found. Although Argireline presented with low toxicity, it demonstrated far lower efficacy than the botulinum toxin and, therefore, is deemed not to be an alternative treatment. 

## Notes

### Acknowledgments

The author would like to thank CNC cosmetic GmbH Philippsburg for producing the skin serum with hyaluronic acids and adding Argireline into half of the products. Further I would like to thank Dr. H. Hofheinz for organizing the provision of the samples in the containers used in this study and the staff of the Klinik am Rhein for their support in recruiting participants, organizing their clinic visits, and providing participant care throughout the study. Finally, the author would like to acknowledge Dr. Wolfgang Reimers for his statistical advice in the planning of the study and his support in the analysis of the data.

### Ethical approval

All procedures performed in the study were performed in accordance with the ethical standards of the institutional and national research committee, as well as the 1964 Helsinki Declaration and its later amendments and other comparable ethical standards.

### Competing interests

The author declares that she has no competing interests. There was no influence from the companies involved in the conduct of the study nor in the analysis or publication of the results. 

## Figures and Tables

**Table 1:**
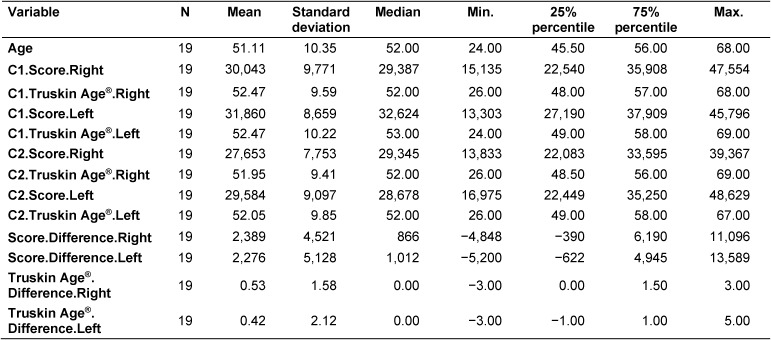
Continuous variables for all participants

**Table 2:**
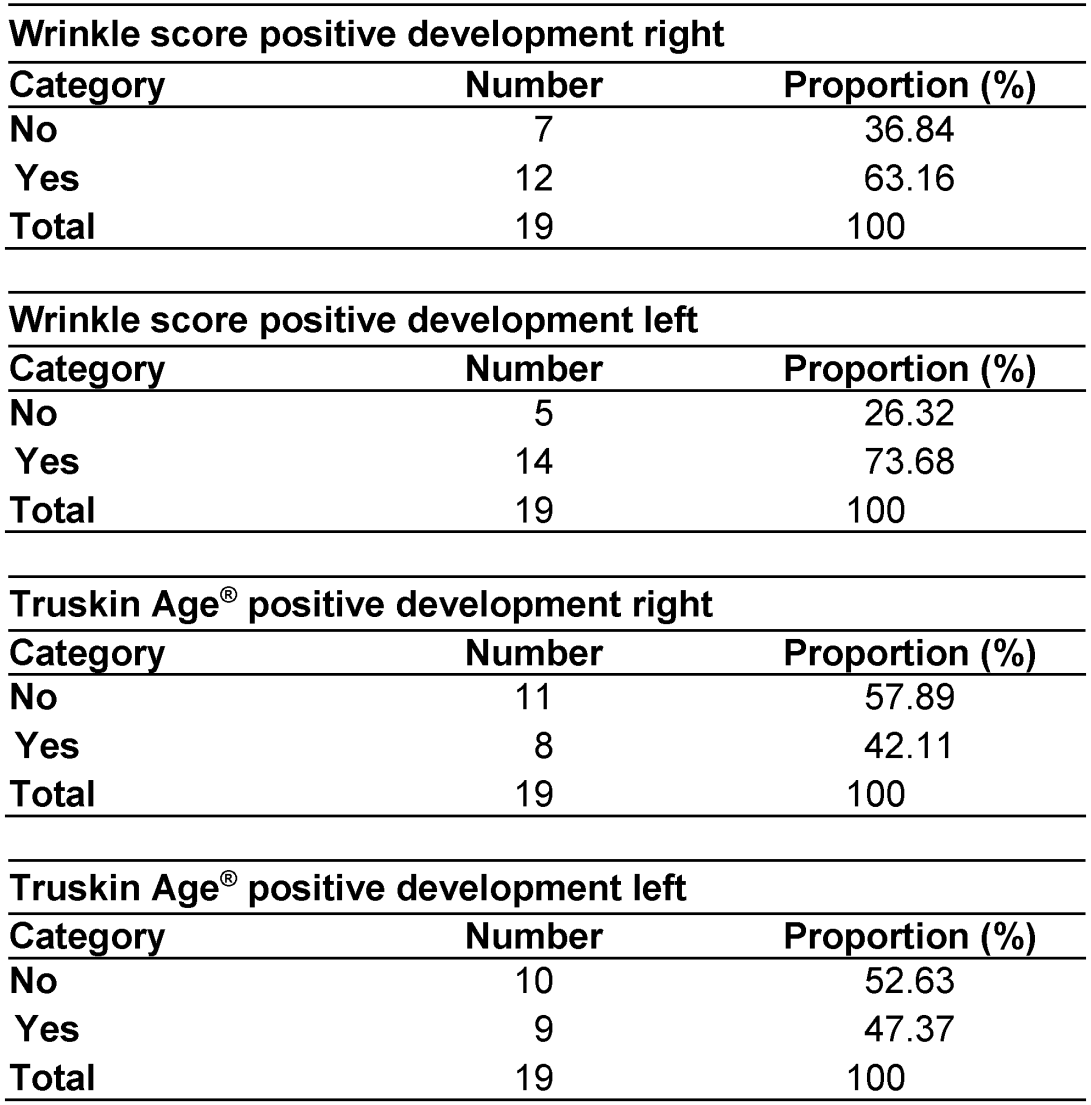
Frequency distribution of the discrete variables

**Table 3:**
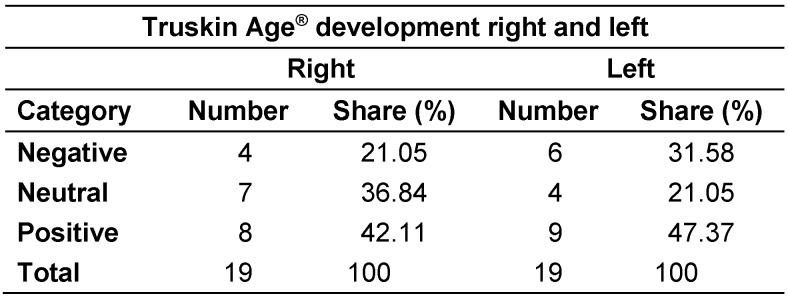
Proportion of negative, neutral, and positive developments in Truskin Age^®^ measurements between time points C1 and C2

**Table 4:**
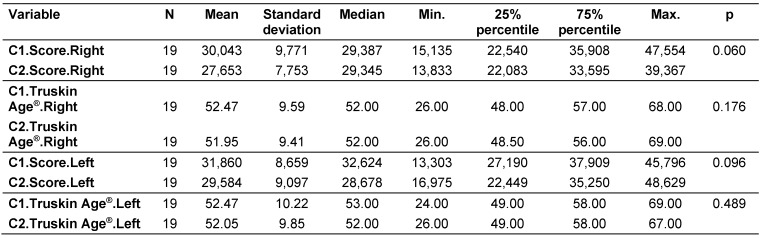
Comparison between C1 and C2 regarding wrinkle and Truskin Age^®^ scores for the right and the left sides of the face

**Table 5:**
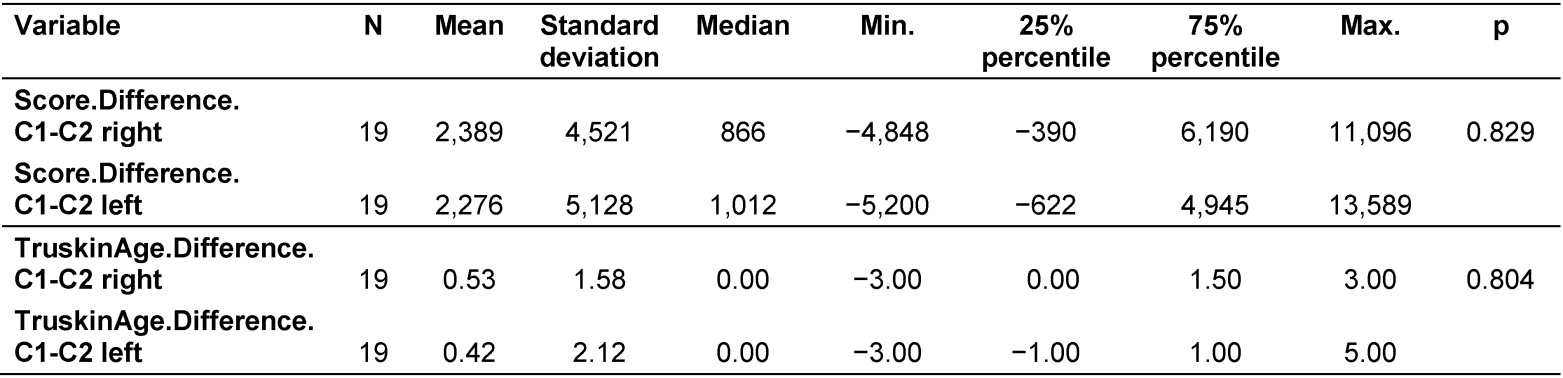
Comparison of the left and right facial side differences of the wrinkle and Truskin Age^®^ scores

## References

[1] Blanes-Mira C, Clemente J, Jodas G, Gil A, Fernández-Ballester G, Ponsati B, Gutierrez L, Pérez-Payá E, Ferrer-Montiel A (2002). A synthetic hexapeptide (Argireline) with antiwrinkle activity. Int J Cosmet Sci.

[2] Ruiz MA, Clares B, Morales ME, Cazalla S, Gallardo V (2007). Preparation and stability of cosmetic formulations with an anti-aging peptide. J Cosmet Sci.

[3] Lungu C, Considine E, Zahir S, Ponsati B, Arrastia S, Hallett M (2013). Pilot study of topical acetyl hexapeptide-8 in the treatment for blepharospasm in patients receiving botulinum toxin therapy. Eur J Neurol.

[4] Wang Y, Wang M, Xiao S, Pan P, Li P, Huo J (2013). The anti-wrinkle efficacy of argireline, a synthetic hexapeptide, in Chinese subjects: a randomized, placebo-controlled study. Am J Clin Dermatol.

[5] Daniell HW (1971). Smoker’s wrinkles. A study in the epidemiology of “crow’s feet”. Ann Intern Med.

[6] Doshi SN, Alster TS (2005). Combination radiofrequency and diode laser for treatment of facial rhytides and skin laxity. J Cosmet Laser Ther.

[7] Henseler H (2022). Validation of the Visia Camera System for skin analysis through assessment of the correlations among the three offered measurements - the percentile, feature count and absolute score - as well as the three capture perspectives, from the left, front and right. GMS Interdiscip Plast Reconstr Surg DGPW.

[8] Henseler H (2022). Investigation of the precision of the Visia complexion analysis camera system in the assessment of skin surface features. GMS Interdiscip Plast Reconstr Surg DGPW.

[9] Hedderich J, Sachs J (2020). Angewandte Statistik.

[10] Ackermann H (2022). BiAS für Windows. Programmsystem zur statistischen Datenanalyse, Version 11.12.

[11] Henseler H, Smith J, Bowman A, Khambay BS, Ju X, Ayoub A, Ray AK (2013). Subjective versus objective assessment of breast reconstruction. J Plast Reconstr Aesthet Surg.

[12] Henseler H, Hille-Betz U, Vogt PM (2015). Validierung subjektiver Schätzungen des weiblichen Brustvolumen und Vergleich zur objektiven Methode. Handchir Mikrochir Plast Chir.

[13] Henseler H, Hille-Betz U, Vogt PM (2016). Estimation of breast implant volumes: error assessment of the subjective judgment method. GMS Ger Plast Reconstr Aesthet Surg.

[14] Grosicki M, Latacz G, Szopa A, Cukier A, Kieć-Kononowicz K (2014). The study of cellular cytotoxicity of argireline - an anti-aging peptide. Acta Biochim Pol.

[15] Krishnan G, Roberts MS, Grice J, Anissimov YG, Moghimi HR, Benson HA (2014). Iontophoretic skin permeation of peptides: an investigation into the influence of molecular properties, iontophoretic conditions and formulation parameters. Drug Deliv Transl Res.

[16] Hoppel M, Reznicek G, Kählig H, Kotisch H, Resch GP, Valenta C (2015). Topical delivery of acetyl hexapeptide-8 from different emulsions: influence of emulsion composition and internal structure. Eur J Pharm Sci.

[17] Kraeling ME, Zhou W, Wang P, Ogunsola OA (2015). In vitro skin penetration of acetyl hexapeptide-8 from a cosmetic formulation. Cutan Ocul Toxicol.

[18] Raikou V, Varvaresou A, Panderi I, Papageorgiou E (2017). The efficacy study of the combination of tripeptide-10-citrulline and acetyl hexapeptide-3. A prospective, randomized controlled study. J Cosmet Dermatol.

[19] Lim SH, Sun Y, Thiruvallur Madanagopal T, Rosa V, Kang L (2018). Enhanced Skin Permeation of Anti-wrinkle Peptides via Molecular Modification. Sci Rep.

[20] Palmieri B, Noviello A, Corazzari V, Garelli A, Vadala M (2020). Skin scars and wrinkles temporary camouflage in dermatology and oncoesthetics: focus on acetyl hexapeptide-8. Clin Ter.

[21] McGuinn KP, Ross NA, Wang JV, Saedi N (2020). Combination Tripeptide/Hexapeptide Serum with 1540 nm Nonablative Fractional Laser for the Treatment of Striae Distensae: A Pilot Study. Skinmed.

[22] Kluczyk A, Ludwiczak J, Modzel M, Kuczer M, Cebrat M, Biernat M, Bąchor R (2021). Argireline: Needle-Free Botox as Analytical Challenge. Chem Biodivers.

[23] Chen CF, Liu J, Wang SS, Yao YF, Yu B, Hu XP (2021). Mycobacterium abscessus infection after facial injection of argireline: A case report. World J Clin Cases.

[24] Mestre R, García N, Patiño T, Guix M, Fuentes J, Valerio-Santiago M, Almiñana N, Sánchez S (2021). 3D-bioengineered model of human skeletal muscle tissue with phenotypic features of aging for drug testing purposes. Biofabrication.

[25] Nagendran ST, Ali MJ, Dogru M, Malhotra R (2022). Complications and adverse effects of periocular aesthetic treatments. Surv Ophthalmol.

